# Impact of Bioactive Compounds of Plant Leaf Powders in White Chocolate Production: Changes in Antioxidant Properties during the Technological Processes

**DOI:** 10.3390/antiox11040752

**Published:** 2022-04-10

**Authors:** Szymon Poliński, Patrycja Topka, Małgorzata Tańska, Sylwia Kowalska, Sylwester Czaplicki, Aleksandra Szydłowska-Czerniak

**Affiliations:** 1 Department of Analytical Chemistry and Applied Spectroscopy, Faculty of Chemistry, Nicolaus Copernicus University in Toruń, 87-100 Toruń, Poland; szymon.polinski@kopernik.com.pl (S.P.); skowalska@umk.pl (S.K.); 2 Confectionery Factory “Kopernik” S.A., 87-100 Toruń, Poland; patrycja.topka@wp.pl; 3 Department of Food Plant Chemistry and Processing, Faculty of Food Sciences, University of Warmia and Mazury, 10-718 Olsztyn, Poland; sylwester.czaplicki@uwm.edu.pl

**Keywords:** matcha, moringa, bioactive compounds, white chocolate, processing, antioxidant properties

## Abstract

Bioactive compounds present in the powdered leaves of matcha green tea (*Camellia sinensis* L.) (MGTP) and moringa (*Moringa oleifera*) (MOLP) seem to be related to health benefits due to their antioxidant properties. The growing accessibility of these powders has led to their being more widely used in food production. The aim of this study was to evaluate the total phenolic content (TPC) and antioxidant capacity (AC) of white chocolate (WCh) supplemented with MGTP and MOLP. AC was determined by 2,2-diphenyl-1-picrylhydrazyl (DPPH), 2,2′-azino-bis(3-ethylbenzothiazoline-6-sulfonic acid) (ABTS), cupric ion-reducing antioxidant capacity (CUPRAC), and ferric-reducing antioxidant power (FRAP) assays, whereas TPC was determined by the Folin–Ciocalteu (FC) method. Both additives were incorporated at four levels (1, 2, 3 and 4%) in two chocolate processing steps (conching and tempering). Additionally, the amounts of phenolic acids, tocopherols, and carotenoids in WCh samples enriched by MGTP and MOLP were determined to explain their influence on AC. The results showed that the chocolates supplemented with MGTP were characterized by higher antioxidant properties than those with MOLP. In turn, MOLP significantly increased the content of lipophilic antioxidants in chocolates, tocopherols and carotenoids, which also exhibit pro-health effects. Furthermore, the incorporation of these additives during the tempering process was more relevant to the improvement of the antioxidant properties of WCh.

## 1. Introduction

Many nutritious plants are valuable sources of natural components that improve human health and well-being. In recent years, the nutritional and medicinal properties, such as antioxidant, antimicrobial, anti-inflammatory, antihypertensive, diuretic, antidiabetic, antihyperlipidemic, antineoplastic, antipyretic, antiulcer, cardioprotectant, and hepatoprotectant properties, of *Camellia sinensis* and *Moringa oleifera* plants have been widely reported [1,2].

Matcha (*Camellia sinensis* L.) is a powdered type of Japanese green tea, particularly rich in bioactive components, such as phenolic compounds, which constitute even up to 30% of dry mass (phenolic acids, flavonoids, especially flavans, also known as tea catechins, and tannins), alkaloids (methylxanthines, mainly caffeine) and amino acids (theanine, gamma amino butyric acid) [1]. Moringa (*Moringa oleifera*) is a medicinal Indian herb the leaves of which are a good source of phenolic acids, flavonoids, essential amino acids, minerals (calcium, potassium, magnesium) and fiber [2]. The bioactive compounds present in matcha green tea powder (MGTP) and moringa leaf powder (MOLP) with antioxidant, chemopreventive and mood-enhancing activities play an important role in human health due to the prevention of chronic degenerative diseases. Furthermore, antioxidant compounds contained in MGTP and MOLP have become important in the food industry. Food products supplemented with them have better physicochemical and organoleptic characteristics and also have extended shelf lives. Natural antioxidants of MGTP and MOLP used as additives in various food products act with different functions (complexation with proteins and carbohydrates, inhibition of lipid oxidation and microbiological growth) through interaction/molecular modification mechanisms, enabling the development of innovative, healthy, nutritious and long shelf-life products with enhanced sensory acceptance [2].

Different studies have attracted great interest in the application of bioactive substances in the creation of functional foods. The effects of powdered leaves and extracts of *Camellia sinensis* L. and *Moringa oleifera* on the physicochemical and sensory quality characteristics of bakery [3,4], confectionery [5,6], dairy [7,8] and meat [9,10] products, beverages [11,12], noodles [13,14], oils and fat-based [15,16] products have been widely studied in recent years. Furthermore, incorporating bioactive compounds from tea and moringa powders and extracts into different types of chocolate has enhanced their antioxidant and antimicrobial activities, sensory acceptance, and shelf life through inhibition of lipid oxidation [17,18,19,20,21,22,23,24]. White chocolate (WCh) samples have also been supplemented with freeze-dried acai powder [25], cinnamon oleoresin [26] and cinnamon essential oil [27].

It is known that chocolate is one of the world’s most popular sweets [28]. Different types of chocolate (e.g., dark, milk and white) can be found on the market, usually made by combining cocoa mass (cocoa powder, cocoa butter and/or cocoa liquor) with sugar and other additives in varying quantities. Among chocolate products, dark chocolate is the most desirable because it contains significantly more health-promoting polyphenols than milk chocolate. In turn, WCh has been regarded as an even unhealthier product than other types of chocolate because fat-free cocoa solids are omitted [18]. The production process of chocolate is unique and complicated and consists of six main stages, including mixing, refining, conching, tempering, molding and cooling. Among these, conching and tempering were identified as the key processes in chocolate manufacturing that affect the final characteristics of chocolate [29]. Conching consists of mixing, shearing and aerating the chocolate mass during heating at a specific temperature (>40 °C). This process contributes to the development of the viscosity, texture and flavor of chocolate [30]. Tempering is a controlled cocoa butter crystallization technique (at temperatures up to 32 °C) that helps stabilize the polymorphic transitions of cocoa butter crystals during storage. Tempering is a crucial phase in chocolate manufacture since it influences perception by consumers and provides the smooth and shiny appearance of chocolate [31].

However, the final content of bioactive compounds and the antioxidant potential of chocolate are a function of several variables, some related to the raw materials and others related to processing and formulation [32,33,34]. The increase in antioxidant properties during chocolate production, especially in white and milk chocolate types, has been investigated by many researchers. Although some researchers have successfully introduced matcha and moringa into WCh products [17,24,35], there are no results from testing of the supplementation stage on chocolate antioxidant properties. Literature data show that both additives mainly increase the content of phenolic compounds in chocolate products [17,18,24,35]. Their mechanism of antioxidant actions in food products is either by hydrogen atom transfer (HAT), single electron transfer (SET), sequential proton loss electron transfer (SPLET) or transition metal chelation (TMC) [36]. The 2,2-diphenyl-1-picrylhydrazyl (DPPH) radical scavenging activity assay is the most extensively used to evaluate the antioxidant activity of foods rich in phenolic compounds, measuring their ability to act as free radical scavengers or hydrogen donors. A strong correlation between antioxidant activity measured by the DPPH assay and the content of phenolic compounds in chocolate has been confirmed by many researchers, e.g., [17,24,35,37]. However, MGTP and MOLP can provide lipophilic antioxidants such as carotenoids and tocopherols which also affect the antioxidant properties of the enriched products [1,38,39,40].

The main objective of this study was to investigate for the first time the effect of the incorporation of MGTP and MOLP at four different concentrations into chocolate samples during two processes (conching and tempering) on the total phenolic content (TPC) and antioxidant capacity (AC) of the fortified products. Antioxidant properties of two plant leaf powders and enriched chocolate samples were determined spectrophotometrically by using four modified analytical methods: DPPH, 2,2′-azino-bis(3-ethylbenzothiazoline-6-sulfonic acid) (ABTS), cupric reducing antioxidant capacity (CUPRAC), and ferric reducing antioxidant power (FRAP), while TPC was analyzed by Folin–Ciocalteu (FC) assay. Additionally, the amounts of individual hydrophilic (phenolic acids) and lipophilic (tocopherols, carotenoids) antioxidants in leaf powders and chocolates supplemented with them were determined, and their impact on AC was examined.

## 2. Materials and Methods

### 2.1. Chemicals

Standard phenolic acids (purity > 97%), including caffeic, chlorogenic, *m*-coumaric, *p*-coumaric, ellagic, ferulic, gallic, gentistic, *p*-OH-benzoic, protocatechuic, salicylic, sinapic, syringic, vanillic acids and standard tocopherols (purity > 96%), including α-tocopherol, γ-tocopherol and δ-tocopherol, β-apo-8′-carotenal (purity > 96%), 6-hydroxy-2,5,7,8-tetramethylchromane-2-carboxylic acid (Trolox, TE), 2,2-diphenyl-1-picrylhydrazyl (DPPH), 2,2′-azino-bis(3-ethylbenzothiazoline-6-sulphonic acid) (ABTS), 2,4,6-tris(2-pyridyl)-s-triazine (TPTZ, 99%), ammonium acetate, copper(II) chloride, dichloromethane, hydrochloric acid, iron(III) chloride hexahydrate, neocuproine and HPLC-grade solvents, such as acetonitrile, formic acid, iso-propanol, methanol, methyl tert-butyl ether and *n*-hexane, were purchased from Sigma Aldrich (Poznań, Poland). Analytical-grade reagents, such as acetic acid, acetone, diethyl ether, Folin–Ciocalteu (FC) reagent, methanol, potassium hydroxide, sodium carbonate and sodium sulfate were supplied by Chempur (Piekary Śląskie, Poland).

### 2.2. Materials

Plain white chocolate (WCh) with 33.6% total fat content was produced through a classical technological process in the chocolate factory BARRY CALLEBAUT BELGIUM NV–Aalstersestraat 122–9280 Lebbeke Wieze (Belgium).

WCh samples were supplemented with: matcha (*Camellia sinensis* L.), green tea powder (MGTP) produced by BioPlanet (batch number: 287-490520; use-by date: 31 December 2021; country of origin: China), and moringa (*Moringa oleifera*) leaf powder (MOLP) produced by Targoch (batch number: 100927; expiry date: 31 March 2021; country of origin: India).

### 2.3. Preparation of Chocolates Supplemented with MGTP and MOLP

Chocolate samples were produced under industrial conditions in the Confectionery Factory Kopernik S.A., Toruń, Poland. A chocolate-tempering machine, Pomati T35, with a mold-filling device (Codogno LO, Italy) was used to prepare WCh enriched with different amounts of MGTP and MOLP. The additives were introduced to the WCh during two processes, conching (Co) and tempering (Te).

First, 50 kg of WCh was melted at 45 °C in the chocolate heating and mixer tank Pomati 150 (Codogno LO, Italy). In the case of samples with supplementation during the Co process, the additives were added to the liquid chocolate. The WCh was homogenized with the addition of MGTP and MOLP for 5 h in the tank of the Pomati 150 (a simulation of the liquid phase of the Co process). The next step was the Te process. The chocolate mass was then poured into the Pomati T35 with a mold-filling device, and it was circulated between the sharpener tank and the coating belt for the dispensing of the chocolate directly into the molds. The Pomati T35 with a mold-filling device was switched to cooling mode, and the temperature was set to 29 °C, while the temperature on the mold-filling device was 0.5 °C higher.

**Figure 1:**
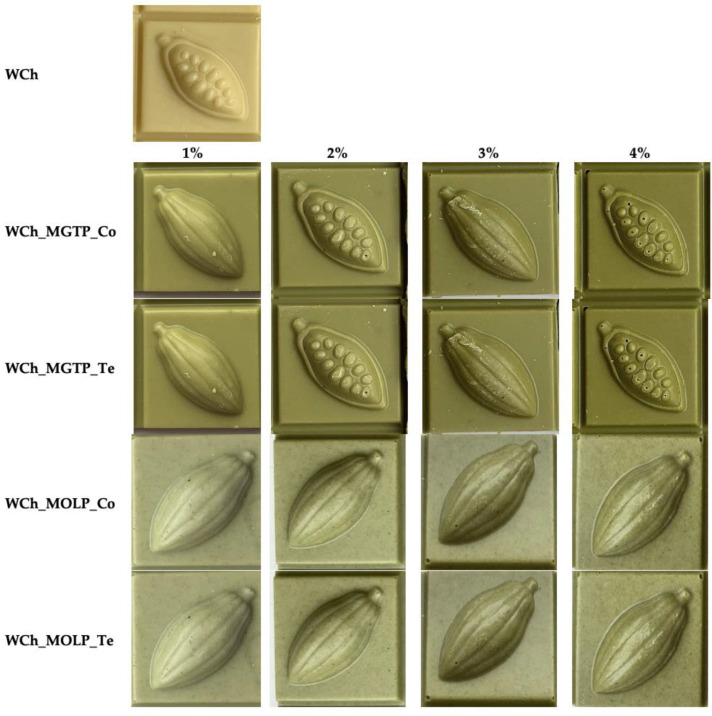
Photographs of white chocolate (WCh) samples without and with different amounts (1–4%) of matcha green tea powder (MGTP) and moringa leaf powder (MOLP) introduced during the conching (Co) and tempering (Te) processes.

In the case of samples with supplementation during the Te process, the additives were mixed with the liquid chocolate for 10 min before it was poured into the molds. The pre-crystallized chocolate mass was then poured into 10 × 10 g plastic molds and cooled in a cooling tunnel (Kreüter, Kühlkanal Universal K.K. 1050, Hamburg, Germany) at 10 °C for 20 min. The samples were then packaged and stored in a dark and dry place at 18 °C until the analysis. The same procedure was carried out to produce chocolate samples enriched with MGTP and MOLP at four different concentrations (1, 2, 3 and 4%). The research material consisted of 17 chocolate samples (Figure 1). The control sample was plain white chocolate (WCh) without the addition of any leaf powder, and 16 supplemented chocolate samples were differentiated by additive type (MGTP, MOLP), amount (1, 2, 3, 4%) and supplementation stage (Co, Te).

### 2.4. Extraction of Phenolic Antioxidants from Leaf Powders

Extraction of antioxidants from MGTP and MOLP was performed using an ultrasonic water bath (5200DTD, Chemland, Stargard Szczeciński, Poland) equipped with a digital timer and temperature controller, at a power and frequency of 180 W and 40 kHz, respectively. Exactly 0.5 g of each powder was mixed with methanol (70%, *v*/*v*; 25 mL) in Erlenmeyer flasks, stirred and placed in an ultrasonic bath. Water in the ultrasonic bath was circulated and regulated at a constant temperature (25 ± 0.3 °C) to avoid water temperature increases as a result of exposure to ultrasound. Each sample was sonicated in duplicate for 10 min and centrifuged at 1880× *g* for 15 min (centrifuge MPW-54, Chemland, Stargard Szczeciński, Poland).

### 2.5. Extraction of Phenolic Antioxidants from Chocolate Samples

Chocolate extracts were prepared using a conventional extraction technique as previously described by Adamson et al. [41] with some modifications. The ground chocolate samples (2.0 g) were weighed on an analytical balance and placed in a dry ground glass joint surface flask with a capacity of 100 mL. Then, 10 mL of methanol (70%, *v*/*v*) was added, and the mixture was shaken mechanically for 30 min. The procedure was repeated twice, and the obtained extracts were combined and filtered to give 20 mL of extract for analysis. The same procedure was used for each of the tested chocolates.

### 2.6. Determination of Total Phenolic Content in Leaf Powders and Chocolate Samples

The modified, previously described FC method [42] was used to determine TPC in extracts of phenolic compounds obtained from the used leaf powders and tested chocolates. Briefly, 0.25–0.5 mL of extracts were transferred into a 25 mL calibrated flasks, then 0.5 mL of FC reagent was added, and the mixtures were shaken for 3 min. Next, 1 mL of a saturated sodium carbonate solution was added and made up to the mark with distilled water. After 45 min, the solution was centrifuged at 1880× *g* for 5 min in a laboratory centrifuge MPW-54 and absorbance at 765 nm was measured against a reagent blank using a Hitachi U-2900 UV-VIS spectrophotometer (Tokyo, Japan). A calibration curve: A_765_ = (0.1034 ± 0.0025)c_GA_ + (0.0814 ± 0.0147) was prepared for the working solutions of gallic acid (GA) in the concentration range of 0.35–10.51 μg/mL. TPC values were expressed as mg GA equivalents per 100 g of sample.

### 2.7. Determination of Phenolic Acid Content in Leaf Powders and Chocolate Samples

The phenolic acid contents were determined by the RP-HPLC technique according to the method described by Skrajda-Brdak et al. [43]. Briefly, 5 mL of prepared extract of phenolic compounds was evaporated to dryness at temperatures below 50 °C in an R-210-type Büchi vacuum evaporator (Büchi Labortechnik, Flawil, Switzerland). The residue was dissolved in 20 mL of deionized water and acidified to pH 2. Then, phenolic acids were extracted 5 times with 20 mL of diethyl ether and the collected extracts were evaporated in a vacuum evaporator. The dry extract was re-dissolved in 1 mL of methanol and subjected to chromatographic separation on an Agilent Technologies (Santa Clara, CA, USA) 1200 series system fitted out with a photodiode detector with a Waters XBridge C18 column (Milford, MA, USA) (150 mm × 2.1 mm, 3.5 μm) at 30 °C. A gradient elution program was employed, using two elution solvents: solvent A (water/formic acid = 99.85/0.15, *v*/*v*) and solvent B (acetonitrile/formic acid = 99.85/0.15, *v*/*v*). The flow rate was 0.5 mL/min with a gradient elution program as follows: 0–3 min, 99% A; 3–15 min, 99–90% A; 15–25 min, 90–40% A; 25–27 min, 40–20% A; 27–30 min, 20% A; 30–33 min, 20–99% A, and was stable until 40 min. The detection was performed at wavelengths of 260, 280 and 320 nm. Phenolic acids were identified by comparison with the absorption spectra of the reference phenolic acids and their contents were determined from calibration curves of reference standards. The LOD and LOQ were 0.025–0.05 μg/mL and 0.08–0.17 μg/mL, respectively.

### 2.8. Determination of Lipophilic Antioxidants in Leaf Powders and Chocolate Samples

The content of tocopherols was determined according to the method described by Mikołajczak et al. [44]. Each sample of leaf powder (2 g) and chocolate (1 g) was extracted 3 times with *n*-hexane. The collected extracts were evaporated to dryness at temperatures below 50 °C in a R-210-type Büchi vacuum evaporator. Then, the residue was re-dissolved in 5 mL of *n*-hexane and subsequently centrifuged (10 min, 25,000× *g*) in a 5417R-type Eppendorf centrifuge (Eppendorf AG, Hamburg, Germany). The resultant solution was analyzed using a HPLC Agilent Technologies 1200 chromatograph (Santa Clara, CA, USA) equipped with a fluorescence detector from the same company and a LiChrospher Si 60 column (250 mm × 4 mm × 5 μm, Merck, Darmstadt, Germany). A 0.7% solution of iso-propanol in *n*-hexane was used as the mobile phase at a flow rate of 1 mL/min. The fluorescence detector was set at excitation and emission wavelengths of 296 nm and 330 nm, respectively. Tocopherols were quantified using standards of α-, γ- and δ-tocopherols, and their contents were calculated using external calibration curves. The repeatability for α, γ- and δ-tocopherol determinations (expressed as a coefficient of variation) was 2.5%. LOQ was 0.2–0.45, μg/g, while linearity of the calibration curve was confirmed in the range of 0.02–16 μg/mL.

The content of carotenoids was determined according to the method described by Czaplicki et al. [45]. Each sample of leaf powder (1 g) and chocolate (2 g) was diluted in 10 mL *n*-hexane, and β-apo-8′-carotenal as an internal standard and a 40% potassium hydroxide solution in methanol were added. The mixture was shaken in a Multi Rotator RS-60 (Biosan, Riga, Latvia) in the dark at room temperature for 16 h. After saponification, 10% sodium sulfate was added, and carotenoids were extracted 5 times with *n*-hexane. Collected extracts were evaporated on a Büchi R-210 rotary evaporator at 45 °C. The residue was re-dissolved in methanol:dichloromethane mixture (45:55, *v*/*v*) analyzed using an Agilent Technologies 1200 RP-HPLC apparatus, equipped with a diode array detector (DAD), a YMC-C30 chromatography column (150 mm × 4.6 mm, 5 μm) and a YMC-C30 precolumn (10 mm × 4.6 mm, 3 μm) (YMC-Europe GmbH, Dinslaken, Germany). The column temperature was set at 30 °C and methanol (A) and methyl tert-butyl ether (B) gradient was programmed as follows: 0–5 min, 95% A; 5–25 min, 95–72% A; 25–33 min, 72–5% A; 33–60 min, 5–95% A. Carotenoids were identified at 450 nm based on retention times and absorption spectra of carotenoid standards. Their contents were calculated with reference to the internal standard. The repeatability for β-apo-8′-carotenal determination (expressed as a coefficient of variation) was 2.5%. LOQ was 0.05 μg/g of sample, while the linearity of the calibration curve was confirmed in the range of 1–150 mg/L. Calibration curves from 1 to 150 mg/L were obtained by plotting the peak area ratio of analyzed carotene standard to β-apo-8′-carotenal (internal standard) against the ratios of their concentration. The method linearity in the curve concentration range was shown by linear regression coefficients (R^2^) which were above 0.998. Curves were prepared for standard lutein, zeaxanthin, α-carotene and β-carotene. For quantification of luteoxanthin and *cis*-lutein, the curve prepared for lutein was used as well as for quantification of 13-*cis*-β-carotene and the 9-*cis*-β-carotene curve prepared for β-carotene was used.

### 2.9. Determination of Antioxidant Capacity of Leaf Powders and Chocolate Samples

The AC was determined spectrophotometrically using DPPH, ABTS, CUPRAC and FRAP assays according to procedures described previously with some minor modifications [42]. AC values were expressed as millimoles of TE equivalent per 100 g of the studied sample.

#### 2.9.1. DPPH Assay

The DPPH method was used to determine the AC of the extracts of MGTP, MOLP and chocolates. In brief, 0.2–0.5 mL of extracts were added to 1.8–1.5 mL of methanol and 0.5 mL of DPPH methanolic solution (304.0 μmol/L). The obtained mixtures were shaken vigorously and left in darkness for 15 min. The absorbance was measured at 517 nm against a reagent blank (2 mL of methanol and 0.5 mL of DPPH methanolic solution) using a Hitachi U-2900 UV-VIS spectrophotometer in a 1 cm glass cell. The scavenging of DPPH was calculated using Equation (1):

(1) $$\%DPPH_{scavenging}=\frac{A_{control}-A_{sample}}{A_{control}}\times 100\%$$

where: *A*_*control*_—absorbance of DPPH radical with methanol; *A*_*sample*_—absorbance of DPPH radical with extract (or standard solution).

The calibration curve, %*DPPH* = (782.10 ± 5.74)c_TE_ + (4.03 ± 0.40), was prepared using working solutions of TE in methanol between 0.02 and 0.10 µmol/mL.

#### 2.9.2. ABTS Assay

In this procedure, 0.1–0.3 mL of the studied extracts were added to 2.4–2.2 mL of ABTS^•+^ solution (7 mmol/L), and the mixtures were incubated at 30 °C for 5 min. The absorbance was measured at 734 nm against a reagent blank (2.5 mL of ABTS^•+^ solution).

The scavenging of ABTS was calculated using Equation (2):

(2) $$\%ABTS_{scavenging}=\frac{A_{control}-A_{sample}}{A_{control}}\times 100\%$$

where: *A_control_*—absorbance of ABTS radical cation with methanol; *A_sample_*—absorbance of ABTS radical cation with extract (or standard solution).

The calibration curve, %*ABTS* = (405.39 ± 3.40)c_TE_ + (10.38 ± 0.30), was prepared using working solutions of TE between 0.01 and 0.15 μmol/mL.

#### 2.9.3. CUPRAC Assay

In brief, 0.6 mL of each prepared extract, 2 mL of 0.01 mol Cu(II)/L, 2 mL of neocuproine solution (0.0075 mol/L) and 2 mL of ammonium acetate buffer (pH = 7) were transferred into 10 mL volumetric flasks and made up to volume with redistilled water. The obtained solutions were kept at room temperature for 30 min. The absorbance was measured at 450 nm against a reagent blank (2 mL of CuCl_2_, 2 mL of neocuproine solution and 2 mL of ammonium acetate buffer made up to 10 mL with redistilled water).

The calibration curve, A_450_ = (8.22 ± 0.06)c_TE_ − (0.0097 ± 0.0029), was prepared using working solutions of TE in methanol between 0.01 and 0.08 μmol/mL.

#### 2.9.4. FRAP Assay

Briefly, 0.1–0.6 mL of extracts from leaf powders and chocolates and 2 mL of FRAP reagent (100 mL of a 10 mmol/L TPTZ solution in 40 mmol/L HCl, 10 mL of 20 mmol/L FeCl_3_ and 25 mL of 0.1 mol/L acetate buffer, pH 3.6) were transferred into 10 mL volumetric flasks and made up to volume with redistilled water. After 20 min, the absorbance was measured at 593 nm against a reagent blank (2 mL of FRAP reagent made up to 10 mL with redistilled water).

The calibration curve, A_593_ = (51.51 ± 0.42)c_TE_ − (0.0023 ± 0.0040), was prepared using working solutions of TE in methanol between 0.001 and 0.017 μmol/mL.

### 2.10. Chocolate Color Measurements

The color of chocolate samples was measured using equipment for digital image analysis (DIA), including a CCD (charge-coupled device) Nikon DXM-1200 color camera (Nikon Instruments, Melville, USA), a Kaiser RB 5004 HF—High Frequency Daylight Copy Light set (4 × 36 W fluorescent light tubes; color temperature about 5400 K) (Kaiser Fototechnik GmbH & Co. KG, Buchen, Germany) and LUCIA (Laboratory Universal Computer Image Analysis) G v. 4.8 software. The results were expressed in the parameters of the CIEL*a*b* model, and the total difference (ΔE) between a plain sample (*WCh*) and a supplemented white chocolate sample (*WChS*) was calculated using Equation (3):

(3) $$\Delta E=\sqrt{{\left(L^{*}{}_{WCh}-L^{*}{}_{WChS}\right)}^{2\;}+{\left(a^{*}{}_{WCh}-a^{*}{}_{WChS}\right)}^{2\;}+{\left(b^{*}{}_{WCh}-b^{*}{}_{WChS}\right)}^{2\;}}$$

The L* parameter represented lightness, from the darkest black as L* = 0% to the brightest white as L* = 100%. The a* parameter represented green/red color (negative/positive values), while the b* parameter represented blue/yellow color (negative/positive values). The sample color was measured in triplicate on the chocolate surface immediately after production [46].

### 2.11. Sensory Acceptance Test

Experimental chocolates supplemented with 4% MGTP and MOLP during the tempering process were sensorily evaluated. The assessment was made by a trained panel consisting of 12 employees of the Confectionery Factory “Kopernik” S.A., including 9 women and 3 men, aged 24 to 53. All panelists were familiar with sensory analysis techniques and had prior experience of sensory evaluation of chocolate. The panelists were provided with water to rinse their mouths before and after testing each sample. The evaluation was carried out in natural sunlight at room temperature. Firstly, the attributes of each of the tested chocolates were identified. The assessment was discussed by the free-choice profile procedure, in which each individual was asked to describe, using their own terms, the appearance, flavor and texture of the chocolate samples. Then, the features were described according to a five-point rating scale, in which 5 represented extremely desirable quality, 4 desirable quality, 3 good quality, 2 reluctance and 1 product defectiveness.

### 2.12. Data Analysis

All chemical analyses were conducted in triplicate. The results were tested statistically using analysis of variance (ANOVA) followed by Tukey’s test. The effects of the plant powder percentage and supplementation stage were determined using a three-way variance analysis with Wilk’s test. Additionally, the Pearson’s correlation coefficients were calculated for relationships between AC and the content of antioxidants. The analyses were performed using Statistica 13.1 PL software (StatSoft, Kraków, Poland) at *p* ≤ 0.05 significance level.

## 3. Results

### 3.1. Characterization of Antioxidants and the Antioxidant Capacities of Leaf Powders Used for White Chocolate Supplementation

The TPC and HPLC results for leaf powders added to chocolate samples are presented in Table 1. As can be seen, the antioxidant profiles of MGTP and MOLP varied. Although both leaf powders, MGTP and MOLP, were characterized by a high TPC, MGTP was a significantly richer source of polyphenols than MOLP (over 5.5 times higher TPC).

It was found that these plant raw materials contain a number of hydrophilic and lipophilic compounds with antioxidant activity. The hydrophilic antioxidants determined in the leaf powders were phenolic acids, and their higher content (83.33 mg/100 g) characterized MGTP. MOLP contained approximately 3 times fewer of these compounds than MGTP. The predominant phenolic acids in MGTP were chlorogenic acid (45.5 mg/100 g) and gallic acid (21.7 mg/100 g), which in total accounted for 81% of all phenolic acids. Other phenolic acids in this leaf powder were present in smaller amounts, not exceeding 5.5 mg/100 g. MOLP was characterized by a more differentiated phenolic acid profile. In this leaf powder, the contents of three phenolic acids accounted for 61%, including *p*-OH-benzoic acid (9.1 mg/100 g), protocatechuic acid (5.01 mg/100 g) and chlorogenic acid (3.71 mg/100 g). Caffeic, sinapic and *p*-coumaric acids were also present in greater concentrations in MOLP (2–3 mg/100 g).

In contrast, MOLP proved to be a significantly better source of lipophilic antioxidants such as carotenoids (296.5 mg/100 g) and tocopherols (3.31 mg/100 g). MGTP had a more than 3.5 times lower level of carotenoids than MOLP, and the content of tocopherols was only 0.13 mg/100 g of powder. Nine carotenoids in both leaf powders were identified, but the lutein fraction (both forms, *trans* and *cis*) was predominant and accounted for 70% in MOLP and 85% in MGTP. Zeaxanthin and β-carotene were present in a higher amount in MOLP, at 38.57 mg/100 g and 32.6 mg/100 g, respectively. The main tocopherol homologue in MOLP was α-tocopherol, which accounted for 77% of total tocopherols. The levels of γ- and δ-tocopherols were lower, at 0.62 and 0.13 mg/100 g, respectively. In MGTP, the percentage shares of all homologues in the total content of tocopherols were similar.

In order to evaluate the antioxidant properties of the MGTP and MOLP used in this study, two radical scavenging assays (DPPH and ABTS) and two reducing methods (CUPRAC and FRAP) were applied, and the results are presented in Table 1.

It can be noted that the MGTP extract indicated stronger DPPH and ABTS radical scavenging activities than the MOLP extract. The calculated DPPH and ABTS values for MOLP were 8-fold lower than those results for MGTP (Table 1). Furthermore, MOLP extract had 3 and 5 times lower CUPRAC (44.1 mmol TE/100 g) and FRAP (9.65 mmol/100 g) values, respectively, in comparison with MGTP extract (CUPRAC = 153.5 mmol TE/100 g and FRAP = 52.95 mmol TE/100 g). These differences in the AC results, determined by the four analytical methods applied in this study, reflect a difference in the ability of antioxidant compounds in the extracts to quench radicals and reduce ferric and cupric ions. It is noteworthy that the simultaneous determination of lipophilic and hydrophilic antioxidants can be achieved by the CUPRAC and ABTS assays. In turn, the DPPH assay is known to work well with lipophilic antioxidants in alcohol solvents, while the FRAP method is especially appropriate for hydrophilic antioxidants but is not adequate for lipophilic antioxidants [36]. For this reason, high AC values for MGTP extract were related to a high content of phenolic antioxidants in this powder. However, similar DPPH, ABTS and CUPRAC results confirmed a dominant proportion of lipophilic antioxidants in MOLP extract (Table 1).

### 3.2. Phenolic Antioxidants Content in Supplemented White Chocolate

The total amount of polyphenols in the WCh without and with leaf powders added during the Co and Te processes are listed in Table 2.

The control WCh used for the tests had a TPC of 6.84 mg GA/100 g, while supplemented chocolates were characterized by a higher concentration of these compounds. More favorable effects in this respect were observed for chocolates after the addition of MGTP. This additive incorporated during the Co process caused a 7 to 20 times increase in the total content of polyphenols. The same amounts of MOLP added during the chocolate conching resulted in an almost 2 to 6 times increase in the content of these compounds. However, the supplementation of WCh with MGTP and MOLP during the Te process increased TPC by 10–34 times and 2–6 times, respectively.

In this study, a destructive effect of the Co process on the content of polyphenols compared to the Te process was observed. In the case of chocolates supplemented with MGTP, depending on the degree of supplementation, the reduction in the presence of polyphenols was from about 30% to almost 50%. However, the reduction in TPC for chocolates enriched with MOLP was even and amounted to about 5%.

As seen in Table 3, the total content of phenolic acids in WCh was 363.1 μg/100 g.

The predominant phenolic acid in this sample was sinapic acid, which accounted for approximately 66%. Furthermore, the plain WCh contained high amounts of protocatechuic, *p*-OH-benzoic and vanillic acids (21.42–45.35 μg/100 g). Gentistic, chlorogenic, caffeic, ferulic and *p*-coumaric acids were determined in the WCh, but at lower concentrations, not exceeding 12 μg/100 g. The samples of chocolates enriched with MGTP and MOLP powders were characterized by significantly higher contents of phenolic acids than that found in plain WCh. The addition of 4% MGTP increased the total content of phenolic acids in fortified chocolates by more than 8 times, while WCh samples with 4% of MOLP revealed only 3 times higher amounts of total phenolic acids. Chlorogenic and gallic acids predominated in MGTP (45.5 and 21.7 mg/100 g, respectively) as well as in chocolates enriched with this powder (1684–1693 and 797–744 μg/100 g, respectively), regardless of the supplementation stage. In turn, the WCh with 4% MOLP had the highest amounts of *p*-OH-benzoic, sinapic, protocatechuic and chlorogenic acids, ranging between 158.29 and 266 μg/100 g. Unexpectedly, a significantly lower amount of sinapic acid in fortified WCh than in the control sample probably can be explained by the possible chocolate matrix effect due to some macromolecules such as proteins and polysaccharides interacting with this compound present in natural additives and reducing its extractability.

In general, the Te process of WCh supplementation appeared to be more crucial for MGTP, as evidenced by the lesser reduction in phenolic acid content. In the case of MOLP addition, there were no significant differences in the contents of most phenolic acids and total phenolic acids in samples prepared during the Co and Te processes (Table 3, Tukey’s test).

### 3.3. Lipophilic Antioxidants Content in Supplemented White Chocolate

As can be seen in Table 4, the main lipophilic compounds determined by the HPLC in the studied plain WCh were γ-tocopherol (627 μg/100 g), α-tocopherol (105.0 μg/100 g) and *trans*-β-carotene (52.49 μg/100 g).

The addition of 4% MOLP to WCh during the Te process significantly (up to 11,339 μg/100 g) enriched the product with carotenoids. In the WCh_4%MGTP, the content of these compounds was about 4 times lower than in the WCh_4%MOLP, regardless of supplementation stage. However, the MGTP addition increased the content of carotenoids by over 50 times compared to plain WCh. The highest levels of lutein fractions (*cis*- and *trans*-isomers) were found in all enriched chocolates, accounting for 73–86% of all carotenoids. Both leaf powders increased the content of *trans*-β-carotene in WCh by 2 times for MGTP and 25 times for MOLP. It was also found that the addition of MOLP enriched WCh with zeaxanthin (up to 1333 μg/100 g) and *cis*-β-carotene (up to 282 μg/100 g), while the addition of MGTP enriched it with luteoxanthin (up to 161 μg/100 g) and zeaxanthin (up to 99 μg/100 g). In contrast, the total content of tocopherols in the supplemented WCh was lower compared to the plain WCh as a result of their lower levels in leaf powders (Table 1). A slightly higher concentration of α-tocopherol in WCh with MOLP (by about 8%) was noted compared to WCh.

Analyzing the impact of the supplementation stage on the lipophilic antioxidant profile, it was found that generally more carotenoids were retained in the product when both leaf powders were added during the Te process. In the case of tocopherols, varied effects were observed, but most of the differences were not statistically significant.

### 3.4. Antioxidant Capacity of Supplemented White Chocolate

Similarly, as in the case of the leaf powders, the AC of WCh without and with MGTP and MOLP added during the Co and Te processes was evaluated by two radical scavenging assays (DPPH and ABTS) and two reducing potential assays (CUPRAC and FRAP). The obtained DPPH, ABTS, CUPRAC and FRAP results are presented in Table 5.

Plain WCh was characterized by a relatively low AC, from 0.08 mmol TE/100 g (FRAP test) to 0.53 mmol TE/100 g (CUPRAC test). In the enriched chocolates, the addition of 1 to 4% MGTP to WCh during the Co process caused a significant increase in the AC values compared to WCh. The increase was from 5 times for WCh_1%MGTP_Co (CUPRAC assay) to 39 times for WCh_4%MGTP_Co (FRAP assay), whereas the MOLP increased the AC of chocolate samples by up to 6 times (the greatest enhancement was observed for WCh_4%MOLP_Co as determined by DPPH assay). The same amounts of MGTP added to WCh during the Te process increased AC by 5 times for WCh_1%MGTP_Te (CUPRAC assay) up to 57 times for WCh_4%MGTP_Te (DPPH assay), but 4% of MOLP resulted in an AC increase of up to 7 times in the studied samples. A destructive effect of the Co process on the antioxidant properties of WCh incorporated with plant powders was also observed; in the case of chocolate supplemented with MGTP, depending on the degree of supplementation and the analytical method, the reduction in AC ranged from approximately 0% for WCh_1%MGTP analyzed by FRAP assay to almost 50% for WCh_4%MGTP tested by the DPPH method.

### 3.5. Effectiveness of Enrichment and Processing Stages in Increasing the Antioxidant Properties of Supplemented White Chocolate

In Table 6, the effect of different factors (plant powder percentage and the stage of its addition to the chocolate) on the TPC and AC of chocolate is presented. The highest effect of the powder percentage on both chocolate parameters, TPC and AC, was noted (57–100% of the explained variance). The MOLP percentage was especially highly decisive for the TPC and AC of supplemented chocolate (>74% of the explained variance). The MGTP percentage had a greater impact on AC as determined by ABTS, CUPRAC and FRAP assays (> 91% of the explained variance), while TPC and DPPH radical scavenging activity were less dependent on the powder percentage (68.60 and 56.78% of the explained variance, respectively).

The impact of the supplementation stage on the tested parameters was of greater importance for MGTP (22.09 and 31.07% of the explained variance for TPC and AC from the DPPH assay, respectively). However, the addition of MOLP at different processing stages generally had a relatively low or not statistically significant (*p* > 0.05) effect on the antioxidant properties of WCh (Table 6). The exception was only the AC determined by the ABTS assay because 15.02% of the explained variance was noted. It was found that the summed effect of two factors (powder type and supplementation stage) on TPC and DPPH of WCh with MGTP (9–12% of the explained variance) and ABTS of WCh with MOLP (10.48% of the explained variance) was moderate, while for other studied parameters, the influence of the interaction of the two factors was negligible.

### 3.6. Correlations between Antioxidant Capacity and Individual Antioxidants in Supplemented White Chocolate

Regression analysis was performed for correlations between the AC determined by four different analytical methods, TPC analyzed by FC assay and the contents of individual antioxidants in chocolates with MGTP and MOLP added during two technological processes (Table 7).

It can be noted that the TPC results correlated significantly (*p* < 0.05) positively with a total antioxidant potential of studied chocolates determined by DPPH (r = 0.99), ABTS (r = 0.95), CUPRAC (r = 0.95) and FRAP (r = 0.93) methods.

However, moderate correlation coefficients (r = 0.29–0.56) for the relationships between some phenolic acids, such as protocatechuic, *p*-OH-benzoic, caffeic, *p*-coumaric and ellagic acids, and AC were observed. Unexpectedly, there were negative correlations between the contents of sinapic acid and all AC values (r varied from −0.55 to −0.63). This can be explained by the fact that the sinapic acid level in fortified chocolates with the highest AC values was significantly lower than in plain WCh without plant additives, having the lowest antioxidant potential analyzed by all analytical tests (Table 3 and Table 5). It was also found that most phenolic acids and carotenoids in chocolates were more closely correlated with radical scavenging activity as analyzed by DPPH assay. Moreover, low positive correlation coefficients for the relationships between α- and δ-tocopherol and AC (r = 0.03–0.30) were calculated, while there were low negative correlations between γ-tocopherol and AC (r varied from −0.24 to −0.43) and total tocopherol and AC (r varied from −0.14 to −0.35). This fact indicated that tocopherol homologues in chocolate samples were not capable of scavenging DPPH radicals, ABTS radical cations and reducing oxidants (cupric and ferric ions).

### 3.7. Effect of Leaf Powders Addition on Color Parameters and Sensory Properties of White Chocolate

Color is one of the crucial attributes of a product because it attracts customers. It also influences acceptance of the sensory characteristics of a food product [47]. The color measurements confirmed that the composition of chocolate had a significant impact on the intensity of the green color (negative a* values) and that it increased with increasing MGTP and MOLP concentrations in supplemented chocolates (Table 8). 

**Figure 2:**
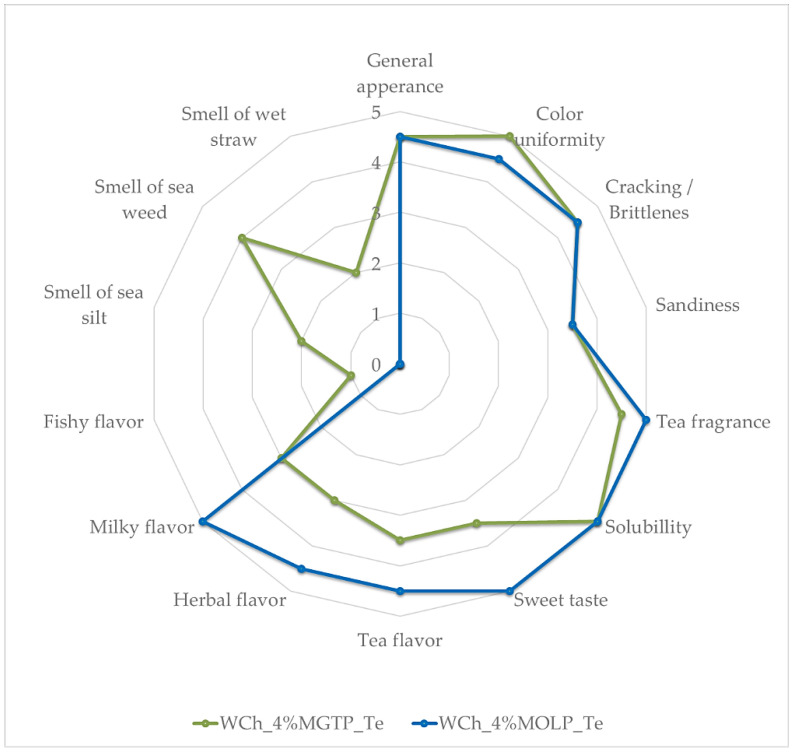
Average sensory ratings of individual attributes of white chocolates with 4% addition of matcha green tea powder and moringa leaf powder introduced during tempering process.

The highest greenness (a* approximately -13) was found in all samples with 4% powdered leaves, regardless of their type and stage of supplementation. Yellowness (positive b* values) was more dependent on the type of additive. It was found that the values of the b* parameter were more variable for MGTP additives (within a range of 18–44), and the highest value was determined in the case of WCh_4%MGTP_Co. Furthermore, all additives decreased the lightness (L* values) from 7.6% (WCh_1%MGTP_Te) to 21.5% (WCh_4%MGTP_Co). In addition, the total color difference (ΔE) parameter which describes a difference between the color of white chocolate before and after supplementation was calculated. All values of ΔE were high and ranged from 9.6–38.5. It is assumed [48] that ΔE > 5 indicates that potential consumers would notice a different color in the case of each of the supplemented chocolates compared to WCh. Interestingly, unlike changes in TPC and AC, the supplementation stage generally did not have a significant effect on chocolate color parameters (*p* > 0.05). Therefore, for the sensory acceptance test, only two samples, WCh_4%MGTP_Te and WCh_4%MOLP_Te, were chosen. Average sensory assessments of individual attributes of chocolates with MGTP and MOLP are presented in Figure 2. In the chocolate supplemented with MGTP, the smell of sea mud, the smell of seaweed, the smell of wet straw and the taste of fish were distinguished, which were not recorded in the case of chocolate with MOLP.

The average score for WCh_4%MGTP_Te was 3.81, while WCh_4%MOLP_Te had a higher sensory score value of 4.74. Chocolate supplemented with MOLP was characterized by a more desirable sensory profile, resulting in the lack of negative discriminants present in chocolate with the addition of MGTP. Chocolate enriched with MOLP was frequently described as having an extremely desirable quality.

The evaluators paid particular attention to the wide range of flavor characteristics of chocolate supplemented with MGTP and unanimously agreed that it would be appreciated by connoisseurs. The sweet and milky taste of WCh was more noticeable in chocolate incorporated with MOLP than in chocolate with MGTP. This suggests that MGTP was more intense in flavor; thus, the flavor of the plain chocolate was masked. The smell of chocolate with the addition of MOLP was assessed positively and the panelists did not notice any unusual aromas. Both chocolates, apart from the assessment of taste and smell, were subjected to an evaluation of physical characteristics, i.e., general appearance, where the focus was on the evaluation of gloss, the correct filling of the mold and even thickness of the chocolate bar, cracking/brittleness, sandiness, color uniformity and spreadability. The quality of the chocolates did not differ from each other; only the uniformity of the color of the chocolate supplemented with MGTP was rated higher than that of the sample with the addition of MOLP. This was probably due to the less powdery quality of moringa leaves in comparison with MGTP. The spreadability of both chocolates was unanimously and approvingly assessed and was due to the same WCh type used to produce the final products.

## 4. Discussion

Vegetable raw materials, cocoa and chocolate are products rich in phenolic compounds; therefore, they have become the subject of many publications concerning the determination of antioxidant properties. Todorowic et al. [49] analyzed several types of chocolate, differing mainly in their cocoa contents. Based on the results obtained by them, it can be concluded that the content of cocoa in chocolate has a relevant impact on the amount of polyphenol compounds and the antioxidant potential resulting from their presence. As the cocoa content increases, the antioxidant potential of chocolate also increases. The total content of polyphenols in plain WCh analyzed in our study was at a low level, not exceeding 10 mg GA/100 g of chocolate. The data available in the literature indicate a higher content of these compounds in this type of chocolate. For example, Genovese and Lannes [50] reported that the phenolic content in WCh commercially purchased in Brazil was 96 mg GA/100 g of chocolate. On the other hand, WCh available on the market in Malaysia, used in the research of Meng et al. [34], contained 126.39 mg of catechin/100 g of chocolate. In our study, by adding powdered matcha green tea and moringa leaves, a significant increase in the concentration of polyphenols was achieved. In particular, the addition of MGTP made it possible to produce chocolate with a high polyphenol content (up to 229 mg GA/100 g), similar to the level of polyphenols in dark chocolates available on the market. Mikołajczak and Tańska [51] stated that the content of polyphenols in dark chocolates available on the Polish market was in the range of 252.38–703.13 mg of catechin/100 g. Nevertheless, dark chocolates available in Malaysia and Brazil were characterized by a higher concentration (up to 700 mg/100 g) of these compounds [34,50].

Unfortunately, the characterization of the phenolic profile of chocolates is very limited. Some reports described the phenolic composition of dark chocolate, focusing on flavan-3-ols as the major class in chocolate phenolic profiles, but only an accurate and comprehensive characterization of the phenolic profile of dark chocolate and the impact of Sakura green tea leaves and turmeric powder were investigated by Martini et al. [19]. Individual phenolic compounds in dark chocolate were identified (158, including 67 for the first time) using liquid chromatography-electrospray ionization mass spectrometry. Therefore, our study revealed for the first time that the addition of MGTP and MOLP as well as technological processes (Co and Te) influenced and modified the hydrophilic and lipophilic antioxidant profiles of WCh, increasing individual antioxidant concentrations and total antioxidant potential.

On the other hand, WCh without additives revealed the weak antioxidant properties determined by analytical tests with different mechanisms, including electron transfer (ET) and mixed-mode (ET/hydrogen atom transfer—HAT) assays. However, the addition of powdered plants to WCh samples resulted in a linear increase in their antioxidant potential. It is noteworthy that MGTP containing high amounts of hydrophilic antioxidants with high total antioxidant potential more effectively increased the antioxidant properties of fortified WCh chocolates than the addition of MOLP richer in lipophilic antioxidants. This diversity was caused by different contents of compounds with antioxidant properties, especially polyphenols, carotenoids and tocopherols, in plant powders added to chocolates. Moreover, the variability of AC values for investigated leaf powders and chocolates fortified with them can be explained by the influences of genetic, environmental and technological factors, which affect antioxidant contents. Undoubtedly, the different mechanisms of the analytical methods applied caused discrepancies between the AC results of natural additives and final products.

For comparison, Afifah and Niwat [52] reported a lower antioxidant potential of moringa compared to green tea. It is worth mentioning that powdered moringa leaves enriched WCh samples mainly with lipophilic antioxidants (carotenoids and tocopherols). Our study also confirmed the enhancement of hydrophilic antioxidants and the total AC of chocolates after MOLP addition. Similarly, TPC (10.20–13.83 mg GA/g), DPPH (EC_50_ = 13.53–8.83 mg/mL) and FRAP (9.32–14.86 mM TE/g) values for dark chocolates increased with increasing the concentrations of added moringa leaf powder (1, 3 and 5%) [24].

In contrast, phenolic acids, rutin, vitamin C, tocopherols, epigallocatechin gallate, epigallocatechin, epicatechin gallate and epicatechin were the most abundant antioxidants present in matcha green tea [53]. Lončarević et al. [18] reported that supplementation of WCh with green tea from 60 to 100 g/kg resulted in a significant increase in total phenolics, antioxidant potential and epigallocatechin-3-gallate content (TPC = 0.41 and 2.20–2.73 g GA/kg, DPPH = 1.22 and 12.55–16.12 mmol TE/kg and EGCG = 0.09 and 0.51–0.62 g/kg for control and fortified chocolates, respectively). Moreover, WCh samples with matcha tea revealed approximately 4–15 times higher TPC, DPPH, ABTS and reducing power values than the plain control chocolate [17].

There are a limited number of studies related to the bioactive compounds changed during chocolate processing. In our study, WCh with leaf powders added during the Co process had lower AC and TPC and total phenolic acids, tocopherols and carotenoids contents. Sulistyowati and Misnawi [54] linked these changes to the elevated temperature during conching. Furthermore, Di Mattia et al. [55] showed that changes in the composition of phenolic compounds were dependent on processing conditions. Furthermore, the oxidation and enzymatic mechanisms during the Co process may also influence the content of polyphenols. As a result of these reactions, complexes between polyphenols, amino acids, peptides and proteins are formed [32]. Nevertheless, the loss of polyphenols during the Te process has not been observed by researchers, but this stage in chocolate processing does not involve high temperatures [56].

## 5. Conclusions

The study confirms that chocolates supplemented with powdered matcha green tea and moringa leaves are products with sophisticated sensory characteristics and positive health properties due to significantly higher concentrations of bioactive polyphenolic compounds than are found in plain WCh. Due to the presence of plant powders, innovative chocolates can be classified as confectionery products having high antioxidant properties.

The supplementation of plain WCh at different stages of the production process caused a significant increase in AC and TPC determined by the modified DPPH, ABTS, CUPRAC, FRAP and FC methods, respectively. It was demonstrated that WCh samples fortified with MGTP had a much higher polyphenol content and, at the same time, much more excellent antioxidant properties than WCh enriched with MOLP. Supplementation of WCh samples with colored and flavored additives also resulted in changes in the color and organoleptic properties of the products. Generally, MOLP had a more beneficial effect on the sensory profile of WCh and a richer flavor bouquet.

In this study, the effect of incorporating two leaf powders during two processes (Co and Te) on phenolic and lipophilic antioxidant contents in WCh was observed for the first time. Although MOLP additives provided a lower amount of polyphenols than MGTP, it provided significantly higher levels of lipophilic antioxidants, mainly carotenoids, which also had strong antioxidant properties. Additionally, it was found that there were relationships between the amounts of leaf powders added and the AC and TPC in supplemented chocolates. Nevertheless, significant positive correlations were calculated between the four analytical methods used to determine AC and TPC. In contrast, the results for AC and concentrations of individual hydrophilic and lipophilic antioxidants did not correlate well.

Furthermore, it is worth noting that the stage of supplementation with leaf powders could also be an important factor in determining chocolate’s pro-health effects. The results obtained in the study showed that the incorporation of powdered plant additives during the Co process had a negative effect on the content of polyphenols and carotenoids and the AC of the finished product.

This work highlights that there is a need for further studies to obtain results useful for chocolate manufacturers not only in terms of obtaining the organoleptic properties preferred by consumers but also in developing products having beneficial effects on consumer health.

## Figures and Tables

**Table 1:** Chemical composition and antioxidant capacity of matcha green tea powder and moringa leaf powder.

Quality Parameter	Leaf Powder	
	MGTP	MOLP
Hydrophilic antioxidants		
TPC (mg GA/100 g)	4556 ± 207	821 ± 40
Phenolic acids (mg/100 g)		
Gallic acid	21.7 ± 0.8	0.93 ± 0.01
Protocatechuic acid	5.4 ± 0.7	5.01 ± 0.11
Gentistic acid	0.35 ± 0.01	<LOD
Chlorogenic acid	45.5 ± 1.6	3.71 ± 0.01
*p*-OH-benzoic acid	1.43 ± 0.04	9.1 ± 0.8
Caffeic acid	1.87 ± 0.24	2.79 ± 0.23
Salicylic acid	1.91 ± 0.07	0.47 ± 0.02
Syringic acid	<LOD	0.48 ± 0.01
Ferulic acid	<LOD	1.52 ± 0.25
*m*-Coumaric acid	1.51 ± 0.08	<LOD
Sinapic acid	2.04 ± 0.29	2.80 ± 0.11
Vanillic acid	1.02 ± 0.15	<LOD
*p*-Coumaric acid	0.51 ± 0.07	2.39 ± 0.10
Ellagic acid	0.01 ± 0.01	0.14 ± 0.01
Total phenolic acids	83.33 ± 0.08	29.3 ± 1.5
Lipophilic antioxidants		
Tocopherols (mg/100 g)		
α-tocopherol	0.04 ± 0.00	2.56 ± 0.02
γ-tocopherol	0.04 ± 0.00	0.62 ± 0.02
δ-tocopherol	0.05 ± 0.00	0.13 ± 0.01
Total tocopherols	0.13 ± 0.00	3.31 ± 0.01
Carotenoids (mg/100 g)		
Luteoxanthin	4.61 ± 0.06	0.93 ± 0.01
*Cis*-lutein	2.98 ± 0.05	0.86 ± 0.01
*Trans*-lutein	64.0 ± 1.6	205.3 ± 0.7
Zeaxanthin	3.05 ± 0.01	38.57 ± 0.26
Cryptoxanthin	0.75 ± 0.01	3.37 ± 0.11
13-*cis*-β-carotene	n.d.	2.81 ± 0.02
α-carotene	0.59 ± 0.00	0.43 ± 0.01
*Trans*-β-carotene	1.57 ± 0.02	32.6 ± 0.4
9-*cis*-β-carotene	0.36 ± 0.01	4.13 ± 0.15
Others	0.77 ± 0.02	7.52 ± 0.26
Total carotenoids	78.7 ± 1.6	296.5 ± 1.0
Antioxidant capacity (mmol TE/100 g)		
DPPH assay	260 ± 7	32.1 ± 1.1
ABTS assay	259.1 ± 1.3	33.5 ± 0.3
CUPRAC assay	153.5 ± 2.0	44.1 ± 0.6
FRAP assay	52.95 ± 0.16	9.65 ± 0.03

**Table 2:** Total phenolic content in white chocolate with matcha green tea powder and moringa leaf powder added during conching and tempering.

Chocolate Sample	TPC	
	Content (mg GA/100 g)	Increase (Times) *
WCh	6.84 ± 0.05 ^a^	-
WCh_1%MGTP_Co	50.9 ± 1.4 ^e^	6.4
WCh_1%MGTP_Te	70.6 ± 1.2 ^f^	9.3
WCh_2%MGTP_Co	90 ± 4 ^g^	12.11
WCh_2%MGTP_Te	109.4 ± 1.7 ^h^	15.0
WCh_3%MGTP_Co	97.2 ± 2.9 ^i^	13.2
WCh_3%MGTP_Te	163.5 ± 0.8 ^j^	22.9
WCh_4%MGTP_Co	134 ± 4 ^k^	18.6
WCh_4%MGTP_Te	229 ± 7 ^l^	32.5
WCh_1%MOLP_Co	11.36 ± 0.59 ^a^	0.7
WCh_1%MOLP_Te	12.80 ± 0.49 ^a^	0.9
WCh_2%MOLP_Co	19.95 ± 0.65 ^b^	1.9
WCh_2%MOLP_Te	20.21 ± 0.09 ^b^	1.9
WCh_3%MOLP_Co	29.21 ± 0.79 ^c^	3.3
WCh_3%MOLP_Te	30.33 ± 0.82 ^c^	3.4
WCh_4%MOLP_Co	39.96 ± 0.47 ^d^	4.8
WCh_4%MOLP_Te	40.88 ± 0.58 ^d^	5.0

* Increase calculated in relation to control sample (WCh). Values presented as mean ± standard deviation; means within the same column with superscripts having different letters (a–l) are significantly different (one-way ANOVA and Tukey’s test, *p* < 0.05).

**Table 3:** Phenolic acids content in white chocolate without and with 4% matcha green tea powder and moringa leaf powder added during conching and tempering.

Phenolic Acid	Content in WCh (μg/100 g)	Content in Supplemented WCh (μg/100 g)			
		4%MGTP_Co	4%MGTP_Te	4%MOLP_Co	4%MOLP_Te
Gallic acid	<LOD	744 ± 41 ^b^	797 ± 28 ^c^	37.3 ± 2.4 ^a^	38.9 ± 1.2 ^a^
Protocatechuic acid	21.42 ± 0.26 ^a^	154 ± 4 ^b^	156 ± 4 ^b^	167 ± 14 ^d^	162 ± 6 ^c^
Gentistic acid	2.09 ± 0.48 ^b^	8.32 ± 0.54 ^c^	10.36 ± 0.73 ^d^	1.78 ± 0.29 ^a^	2.08 ± 0.15 ^b^
Chlorogenic acid	7.00 ± 0.11 ^a^	1693 ± 37 ^e^	1684 ± 41 ^d^	158.29 ± 0.05 ^b^	160.00 ± 0.01 ^c^
*p*-OH-benzoic acid	32.6 ± 1.1 ^a^	49.3 ± 0.8 ^b^	51.87 ± 0.91 ^b^	266 ± 5 ^d^	238 ± 6 ^c^
Caffeic acid	4.05 ± 0.47 ^a^	59 ± 2 ^b^	72 ± 4 ^c^	103.2 ± 2.9 ^d^	107.6 ± 2.0 ^e^
Salicylic acid	<LOD	76 ± 3 ^b^	80.1 ± 1.1 ^c^	14.4 ± 1.5 ^a^	16.6 ± 1.8 ^a^
Syringic acid	<LOD	<LOD	<LOD	19.0 ± 0.6 ^a^	20.47 ± 0.28 ^b^
Ferulic acid	11.38 ± 0.17 ^b^	9.32 ± 0.86 ^a^	10.3 ± 0.6 ^a,b^	53.0 ± 2.8 ^c^	71.2 ± 1.7 ^d^
*m*-Coumaric acid	<LOD	56.6 ± 2.5 ^a^	55.0 ±1.3 ^a^	<LOD	<LOD
Sinapic acid	238 ± 1 ^d^	180.3 ± 2.3 ^b^	189.0 ± 1.2 ^c^	177.44 ± 0.17 ^a^	179 ± 6 ^a,b^
Vanillic acid	45.35 ± 0.26 ^b^	81.2 ± 2.5 ^c^	80.6 ± 2.6 ^c^	25.3 ± 0.7 ^a^	26.3 ± 0.7 ^a^
*p*-Coumaric acid	0.83 ± 0.25 ^a^	12.7 ± 1.4 ^b^	15.8 ± 0.4 ^c^	52.72 ± 0.06 ^d^	52 ± 5 ^d^
Ellagic acid	< LOD	0.42 ± 0.00 ^a^	0.42 ± 0.00 ^a^	4.9 ± 1.3 ^b^	4.64 ± 0.24 ^b^
Total phenolic acids	363.1 ± 1.7 ^a^	3124 ± 71 ^b^	3202 ± 65 ^c^	1080 ± 11 ^d^	1079.6 ± 1.5 ^d^

Values presented as mean ± standard deviation; means within the same row with superscripts having different letters (a–e) are significantly different (one-way ANOVA and Tukey’s test, *p* < 0.05).

**Table 4:** Contents of tocopherols and carotenoids in white chocolate without and with 4% matcha green tea powder and moringa leaf powder added during conching and tempering.

Compound	Content in WCh (μg/100 g)	Content in Supplemented WCh (μg/100 g)			
		4%MGTP_Co	4%MGTP_Te	4%MOLP_Co	4%MOLP_Te
Tocopherols					
α-tocopherol	105.0 ± 1.7 ^c^	77.1 ± 1.4 ^b^	64.7 ± 2.4 ^a^	114.2 ± 2.2 ^d^	113 ± 5 ^d^
γ-tocopherol	627 ± 7 ^c^	486.1 ± 1.8 ^a^	477 ± 5 ^a^	536.6 ± 0.5 ^b^	537 ± 7 ^b^
δ-tocopherol	14.65 ± 0.23 ^a^	14.13 ± 0.13 ^a^	14.3 ± 1.2 ^a^	16.34 ± 0.06 ^b^	16.8 ± 0.6 ^b^
Total tocopherols	746 ± 9 ^e^	577.3 ± 0.6 ^b^	556 ± 6 ^a^	660 ± 8 ^c^	667 ± 12 ^d^
Carotenoids					
Luteoxanthin	<LOD	140 ± 5 ^b^	161 ± 9 ^c^	38.3 ± 0.8 ^a^	39.14 ± 0.11 ^a^
*Cis*-lutein	<LOD	95.0 ± 2.1 ^b^	124.2 ± 0.6 ^c^	33.6 ± 0.4 ^a^	34.6 ± 0.8 ^a^
*Trans*-lutein	<LOD	2285 ± 18 ^a^	2430 ± 11 ^b^	8119 ± 31 ^c^	8297± 74 ^d^
Zeaxanthin	<LOD	73 ± 3 ^a^	99 ± 10 ^b^	1209 ± 18 ^c^	1333 ± 36 ^d^
13-*cis*-β-carotene	<LOD	<LOD	<LOD	97 ± 4 ^a^	108 ± 15 ^b^
α-carotene	<LOD	24 ± 3 ^c^	22.46 ± 0.17 ^c^	12.6 ± 1.0 ^a^	16.0 ± 1.4 ^b^
*Trans*-β-carotene	52.49 ± 0.18 ^a^	93.7 ± 2.5 ^b^	109 ± 9 ^c^	1304 ± 45 ^d^	1337 ± 49 ^d^
9-*cis*-β-carotene	<LOD	13.0 ± 0.9 ^b^	5.7 ± 1.0 ^a^	172 ± 5 ^c^	173 ± 4 ^c^
Total carotenoids	52.49 ± 0.18 ^a^	2724 ± 25 ^b^	2951.8 ± 0.9 ^c^	10986 ± 105 ^d^	11339 ± 80 ^e^

Values presented as mean ± standard deviation; means within the same row with superscripts having different letters (a–e) are significantly different (one-way ANOVA and Tukey’s test, *p* < 0.05).

**Table 5:** Antioxidant capacity of white chocolate with matcha green tea powder and moringa leaf powder added during conching and tempering.

Chocolate Sample	AC Determined by Antioxidant Assays (mmol TE/100 g)			
	DPPH	ABTS	CUPRAC	FRAP
WCh	0.21 ± 0.01 ^a^	0.43 ± 0.01 ^a^	0.53 ± 0.01 ^a^	0.08 ± 0.00 ^a^
WCh_1%MGTP_Co	2.48 ± 0.03 ^f^	3.77 ± 0.06 ^f^	2.39 ± 0.10 ^g,h^	0.92 ± 0.01 ^g^
WCh_1%MGTP_Te	3.65 ± 0.6 ^g^	4.13 ± 0.10 ^f^	2.58 ± 0.09 ^h^	0.92 ± 0.01 ^g^
WCh_2%MGTP_Co	3.91 ± 0.15 ^g^	3.77 ± 0.04 ^f^	3.67 ± 0.12 ^i^	1.65 ± 0.03 ^h^
WCh_2%MGTP_Te	5.37 ± 0.14 ^i^	7.49 ± 0.09 ^g^	5.03 ± 0.09 ^j^	1.69 ± 0.07 ^h^
WCh_3%MGTP_Co	4.40 ± 0.13 ^h^	9.07 ± 0.42 ^h^	7.41 ± 0.18 ^k^	2.10 ± 0.05 ^i^
WCh_3%MGTP_Te	8.72 ± 0.33 ^k^	10.82 ± 0.09 ^i^	8.88 ± 0.18 ^l^	2.24 ± 0.01 ^j^
WCh_4%MGTP_Co	5.93 ± 0.16 ^j^	12.52 ± 0.39 ^j^	9.51 ± 0.14 ^m^	3.13 ± 0.01 ^k^
WCh_4%MGTP_Te	11.85 ± 0.39 ^l^	13.88 ± 0.40 ^k^	10.35 ± 0.15 ^n^	3.18 ± 0.06 ^k^
WCh_1%MOLP_Co	0.42 ± 0.01 ^a,b^	0.51 ± 0.02 ^a,b^	0.99 ± 0.01 ^b^	0.16 ± 0.01 ^b^
WCh_1%MOLP_Te	0.45 ± 0.01 ^a,b^	0.57 ± 0.01 ^a,b^	1.03 ± 0.01 ^b^	0.20 ± 0.00 ^b,c^
WCh_2%MOLP_Co	0.65 ± 0.02 ^b,c^	0.84 ± 0.01 ^a,b,c^	1.41 ± 0.02 ^c^	0.26 ± 0.00 ^c^
WCh_2%MOLP_Te	0.75 ± 0.01 ^b,c^	0.93 ± 0.01 ^a,b,c^	1.56 ± 0.01 ^c,d^	0.27 ± 0.00 ^c^
WCh_3%MOLP_Co	0.91 ± 0.01 ^c,d^	0.99 ± 0.04 ^b,c^	1.83 ± 0.01 ^d,e^	0.37 ± 0.00 ^d^
WCh_3%MOLP_Te	1.00 ± 0.03 ^c,d^	1.58 ± 0.05 ^d^	1.94 ± 0.01 ^e,f^	0.38 ± 0.00 ^d,e^
WCh_4%MOLP_Co	1.26 ± 0.02 ^d,e^	1.30 ± 0.06 ^c,d^	2.22 ± 0.01 ^f,g^	0.46 ± 0.00 ^e^
WCh_4%MOLP_Te	1.50 ± 0.03 ^e^	2.15 ± 0.05 ^e^	2.40 ± 0.01 ^g,h^	0.59 ± 0.01 ^f^

Values presented as mean ± standard deviation; means within the same column with superscripts having different letters (a–n) are significantly different (one-way ANOVA and Tukey’s test, *p* < 0.05).

**Table 6:** Effect of matcha green tea powder and moringa leaf powder percentage and the stage of their addition (percentage of explained variance) on total phenolic content and antioxidant capacity of white chocolate.

Factor	TPC	AC Determined by Antioxidant Assays			
		DPPH	ABTS	CUPRAC	FRAP
MGTP addition					
Powder percentage (PP)	68.60	56.78	91.49	96.59	99.63
Supplementation stage (SS)	22.09	31.07	5.62	2.56	0.12
PP x SS	9.05	11.81	2.59	0.72	0.10
Other factors	n.s.	n.s.	n.s.	n.s.	n.s.
MOLP addition					
Powder percentage (PP)	99.55	96.02	74.15	98.17	93.04
Supplementation stage (SS)	n.s.	2.69	15.02	1.49	3.03
PP x SS	0.20	1.11	10.48	0.30	3.88
Other factors	n.s.	n.s.	n.s.	n.s	n.s.

n.s. = effect not significant; three-way analysis with Wilks’s test (*p* ≤ 0.05).

**Table 7:** Correlation coefficients (r) between antioxidant capacity determined by different analytical methods, total phenolics, phenolic acids, carotenoids and tocopherols in the investigated chocolates.

Compound	DPPH	ABTS	CUPRAC	FRAP
TPC	0.99 *	0.95 *	0.95 *	0.93 *
Gallic acid	−0.10	0.11	0.17	0.17
Protocatechuic acid	0.50	0.49	0.56	0.52
Gentistic acid	−0.19	−0.01	0.05	0.05
Chlorogenic acid	−0.06	0.15	0.21	0.22
*p*-OH-benzoic acid	0.56	0.35	0.35	0.31
Caffeic acid	0.49	0.37	0.42	0.38
Salicylic acid	−0.04	0.16	0.23	0.23
Syringic acid	0.44	0.24	0.24	0.21
Ferulic acid	0.29	0.11	0.11	0.08
*m*-Coumaric acid	−0.10	0.13	0.18	0.19
Sinapic acid	−0.55	−0.57	−0.63	−0.60
Vanillic acid	−0.26	−0.03	0.01	0.03
*p*-Coumaric acid	0.52	0.33	0.35	0.31
Ellagic acid	0.52	0.32	0.33	0.29
Total phenolic acids	0.01	0.21	0.27	0.27
Luteoxanthin	−0.02	0.16	0.22	0.22
*Cis*-lutein	−0.02	0.11	0.19	0.17
*Trans*-lutein	0.52	0.35	0.37	0.33
Zeaxanthin	0.44	0.24	0.25	0.21
13-*cis*-β-carotene	0.42	0.23	0.23	0.19
α-carotene	0.18	0.33	0.41	0.39
*Trans*-β-carotene	0.47	0.27	0.27	0.24
9-*cis*-β-carotene	0.49	0.30	0.31	0.27
Total carotenoids	0.50	0.33	0.35	0.31
α-tocopherol	0.27	0.09	0.04	0.03
γ-tocopherol	−0.24	−0.35	−0.43	−0.41
δ-tocopherol	0.30	0.09	0.09	0.06
Total tocopherols	−0.14	−0.28	−0.35	−0.33

* Statistically significant at *p* ≤ 0.05.

**Table 8:** Color parameters and total color difference (ΔE) of white chocolate with matcha green tea powder and moringa leaf powder added during conching and tempering.

Chocolate Sample	Color parameters			ΔE (-) *
	L* (%)	a* (-)	b* (-)	
WCh	96.9 ± 0.3 ^h^	−3.28 ± 0.06 ^j^	12.83 ± 0.10 ^a^	-
WCh_1%MGTP_Co	88.53 ± 0.19 ^g^	−5.7 ± 0.4 ^i^	18.6 ± 0.4 ^b^	10.4
WCh_1%MGTP_Te	89.47 ± 0.07 ^g^	−5.8 ± 0.4 ^i^	18.35 ± 0.23 ^b^	9.6
WCh_2%MGTP_Co	84.5 ± 0.4 ^f^	−7.9 ± 0.9 ^g,h^	25.1 ± 1.5 ^d,e^	18.0
WCh_2%MGTP_Te	85.23 ± 0.08 ^f^	−7.4 ± 0.3 ^h^	22.9 ± 0.5 ^c,d^	16.0
WCh_3%MGTP_Co	79.82 ± 0.24 ^d^	−11.6 ± 0.5 ^b,c,d^	34.8 ± 0.3 ^g,h^	29.1
WCh_3%MGTP_Te	81.4 ± 0.4 ^e^	−9.4 ± 0.4 ^f,g^	29.56 ± 0.14 ^f^	23.6
WCh_4%MGTP_Co	76.04 ± 0.18 ^a^	−13.4 ± 0.5 ^a^	43.6 ± 1.3 ^j^	38.5
WCh_4%MGTP_Te	76.98 ± 0.29 ^a,b^	−13.1 ± 0.5 ^a,b^	41.9 ± 0.5 ^j^	36.6
WCh_1%MOLP_Co	88.25 ± 0.20 ^g^	−7.1 ± 0.6 ^h,i^	21.6 ± 0.6 ^b,c^	12.9
WCh_1%MOLP_Te	88.83 ± 0.19 ^g^	−6.8 ± 0.4 ^h,i^	20.53 ± 0.09 ^b,c^	11.7
WCh_2%MOLP_Co	84.25 ± 0.20 ^f^	−9.9 ± 0.7 ^e,f^	28.7 ± 1.5 ^f^	21.4
WCh_2%MOLP_Te	84.6 ± 0.5 ^f^	−9.6 ± 0.6 ^f^	27.7 ± 1.2 ^e,f^	20.3
WCh_3%MOLP_Co	80.17 ± 0.24 ^d^	−10.5 ± 0.5 ^d,e,f^	33.0 ± 0.8 ^g^	27.2
WCh_3%MOLP_Te	81.5 ± 0.5 ^e^	−11.3 ± 0.7 ^c,d,e^	33.5 ± 1.5 ^g^	27.0
WCh_4%MOLP_Co	77.73 ± 0.22 ^c^	−12.32 ± 0.16 ^a,b^	38.7 ± 0.8 ^i^	33.4
WCh_4%MOLP_Te	77.21 ± 0.29 ^b,c^	−13.0 ± 0.5 ^a,b^	39.5 ± 1.0 ^i^	34.5

* Calculated in relation to control sample (WCh); values presented as mean ± standard deviation; means within the same column with superscripts having different letters (a–j) are significantly different (one-way ANOVA and Tukey’s test, *p* < 0.05).

## Data Availability

All of the data is contained within the article.
